# Skeleton density and ellipsoid zone loss are prognostic for progression in Macular Telangiectasia Type 2

**DOI:** 10.1038/s41598-024-67801-4

**Published:** 2024-07-27

**Authors:** Lukas Goerdt, Moritz Berger, Julie Jungblut, Jose Luis Rodriguez Garcia, Kristina Pfau, Philipp Herrmann, Frank G. Holz, Maximilian W. M. Wintergerst

**Affiliations:** 1 Department of Ophthalmology, University Hospital Bonn, Ernst-Abbe-Str 2, 53127 Bonn, Germany; 2 Department of Ophthalmology and Visual Sciences, Heersink School of Medicine, University of Alabama at Birmingham, Birmingham, AL USA; 3 Institute for Medical Biometry, Informatics and Epidemiology, Medical Faculty, University of Bonn, Bonn, Germany; 4 Department of Ophthalmology, University Hospital Basel, Basel, Switzerland; 5 Institute of Molecular and Clinical Ophthalmology Basel, Basel, Switzerland; 6 Augenzentrum Grischun, Chur, Switzerland

**Keywords:** Macular Telangiectasia Type 2, Optical coherence tomography angiography, Progression, Longitudinal, MacTel, OCTA, Retinal diseases, Vision disorders

## Abstract

Macular Telangiectasia Type 2 (MacTel) is a chronic, progressive disease of the central retina characterized by vascular and neurodegenerative changes. As there is currently no treatment for non-neovascular MacTel, there is a dearth for biomarkers identifying eyes with an increased risk for disease progression for patient counseling and clinical trial recruitment. Eyes were classified to be stable or progressive, defined by the fundus photography-based grading system by Gass and Blodi. First, structural differences between these two groups were assessed, employing optical coherence tomography (OCT) and OCT-angiography. Univariate regression analyses revealed evidence towards a lower superficial retinal layer (SRL) vessel density (VD), skeleton density (SD) and deep retinal layer (DRL) SD in progressing compared to stable eyes (p = 0.05, p = 0.05, p = 0.07). Second, a multivariable predictive model was employed to examine the predictive value of structural and functional parameters for disease progression. Baseline best corrected visual acuity (BCVA) and SRL SD are prognostic for disease progression (p < 0.001, p = 0.05). The presence of ellipsoid zone (EZ) loss is prognostic for future central retinal thickness (p < 0.01). We propose SRL SD, BCVA, and EZ loss as prognostic biomarkers and as possible outcome measures in future interventional studies in MacTel.

## Introduction

Macular Telangiectasia type 2 (MacTel) is a chronic progressive, bilateral retinal disease, characterized by neurodegenerative and vascular changes. Retinal changes mainly occur temporal to the fovea, predominantly limited to an oval shaped area of 8° horizontal and 5° vertical diameter centered on the fovea^1^. In most cases, MacTel is a slowly progressive disease, ultimately leading to photoreceptor loss and functional impairment^2,3^. The development of secondary neovascularizations (NVs) is a possible and vision threatening complication^4^. To date, there is no effective treatment for patients affected by non-proliferative MacTel. Recently, positive results from a phase 2 clinical trial were published, effectively slowing the progression of area of Ellipsoid Zone (EZ) loss, employing a ciliary neurotrophy factor (CNTF) releasing implant^5^. In case of NV development, the application of intravitreal VEGF inhibitors is indicated^6–8^.

Optical coherence tomography angiography (OCT-A) imaging provides detailed, three-dimensional information about the intraretinal vasculature^9–11^. It has been previously noted that changes of the intraretinal vasculature are apparent in patients affected by MacTel^12–15^. These qualitative changes occur predominantly in the temporal parafovea and include a rarefaction of vessels in the superficial and deep retinal plexus and, in case of retinal-choroidal anastomoses, a proliferation of vessels in the outer retina avascular zone and the outer retinal choriocapillaris (ORCC) layer^12–16^. Earlier investigations described a significant correlation between OCT-A imaging and conventional imaging techniques (e.g. OCT, fluorescein angiography [FLA])^17^. These correlations include a consecutive foveal involvement by retinal alterations starting at the temporal edge and a spatial association of OCT-A alterations with pigment clumps found on color fundus photography (CFP). Quantitative analyses of cross-sectional OCT-A data revealed a decrease of vessel density (VD), skeleton density (SD) and fractal dimension in MacTel eyes compared to age-similar healthy control eyes^13,18^. These findings became more prominent in later disease stages and were more pronounced in the deep compared to the superficial retinal plexus. On the intra-eye level, a VD decrease was noted in the temporal compared to the nasal quadrant^3^. Analyses of the choriocapillaris (CC) revealed a significantly decreased signal intensity, a decreased signal intensity range of the grey-level image, as well as an increase of percentage of nondetectable perfused CC-area (PNPCA) across all MacTel stages compared to controls (all p < 0.001)^13^. Interestingly, no significant changes were observed between different disease stages^13^. Longitudinal OCT-A data in MacTel is limited to date. Demir and colleagues recently reported three cases, who were reviewed over one year. They identified a decrease of VD over time in the deep capillary plexus (DCP) and the superficial capillary plexus (SCP)^19^.

Vekantesh and colleagues described a pre-proliferative disease stage, characterized by high-resolution retinal imaging findings including OCT-A, characterized by a bunching of capillaries in the DCP^20^. All eyes classified as such at baseline developed secondary NVs after the review period. A recent publication investigating OCT-A biomarkers revealed a focal increase in blood flow at the level of the DCP and the ORCC and a formation of retinal-retinal and retinal-choroidal anastomoses preceding the development of NVs^21^. These two findings indicate that OCT-A imaging may hold the potential for predicting disease progression and NV development.

The presented study aims at assessing differences in longitudinally acquired quantitative OCT-A data between stable and progressing eyes. Further, we analyzed the prognostic value of quantitative OCT-A and structural OCT parameters and for the further disease course.

## Results

### Cohort characteristics

A total of 122 eyes of 61 patients (35 female, mean age 62 ± 10 years, mean best corrected visual acuity (BCVA) 0.25 ± 0.26 logMAR; mean BCVA at the last study visit 0.31 ± 0.33 logMAR, mean central retinal thickness (CRT) 236 ± 43 µm, mean CRT 214 ± 44 µm at last visit) were included in this study. Mean follow-up time was 23 months (± 7.8 months) averaging 2.81 ± 1.14 visits. At baseline, no eyes were graded as stage one (0%), 30 eyes as stage two (24.6%), 57 eyes as stage three (46.7%), 20 eyes as stage four (16.3%) and 15 eyes as stage five (12.3%). At the follow up visit, no eyes were graded as stage one (0%), 11 eyes as stage two (9.0%), 58 eyes as stage three (45.9%), 35 as stage four (28.7%) and 18 eyes as stage five (16.4%). In total, 33 eyes underwent a change in Gass and Blodi (G&B) stage between screening and follow-up. 15 eyes converted from stage two to three (45.5%), 4 eyes from two to four (12.1%), 11 eyes from three to four (33.3%) and three eyes from three to five (9%). Out of 345 (total number of visits) G&B disease stage assessments, reader 1 and reader 2 agreed in 330 (95.6%) cases. A third reader was needed for the remaining 15 (4.4%) assessments, which resembles previous results of this group^22^. The descriptive statistics of OCT-A parameters at baseline are presented in Table 1.

**Table 1:** Baseline characteristics of study patients.

Disease stage	N	Age (years)	BCVA (logMar)	CRT (µm)	SRL VD	SRL VDI (pixel)	SRL SD (1/pixel)	DRL VD	DRL VDI (pixel)	DRL SD (1/pixel)	ARL VD	ARL VDI (pixel)	ARL SD (1/pixel)	PNPCA (%)
G&B 1	0	NA	NA	NA	NA	NA	NA	NA	NA	NA	NA	NA	NA	NA
G&B 2	30	62.29 ± 12.2	0.19 ± 0.23	234.89 ± 26.73	0.208 ± 0.045	3,355,802 ± 798,463	6.23E^−8^ ± 3.71E^−9^	0.095 ± 0.025	1,101,921 ± 292,367	8.59E^−8^ ± 2.3E^−9^	0.078 ± 0.32	1,360,454 ± 329,592	5.38E^−8^ ± 1.21E^−8^	73.55 ± 13.59
G&B 3	57	67.36 ± 8.8	0.2 ± 0.24	241.39 ± 31.34	0.216 ± 0.38	3,528,495 ± 754,534	6.21E^−8^ ± 5.07E^−9^	0.092 ± 0.025	1,099,183 ± 305,130	8.44E^−8^ ± 3.43E^−9^	0.064 ± 0.034	1,217,670 ± 396,819	4.78E^−8^ ± 1.34E^−8^	72.64 ± 18.79
G&B 4	20	65.68 ± 12.3	0.31 ± 0.31	220.05 ± 48.95	0.196 ± 0.043	3,221,793 ± 821,202	6.21E^−8^ ± 3.01E^−9^	0.078 ± 0.021	933,024 ± 237,905	8.47E^−8^ ± 2.9E^−9^	0.033 ± 0.026	802,070 ± 354,855	3.51E^−8^ ± 1.37E^−8^	71.64 ± 10.29
G&B 5	15	65.74 ± 11.2	0.43 ± 0.38	250.4 ± 57.58	0.187 ± 0.055	2,954,952 ± 969,589	6.42E^−8^ ± 3.53	0.076 ± 0.031	890,729 ± 362,774	8.65E^−8^ ± 2.7E^−9^	0.018 ± 0.014	614,459 ± 2,060,303	2.5E^−8^ ± 1.07E^−8^	65.99 ± 16.35

Data is presented as means ± standard deviation. *BCVA* best corrected visual acuity, *CRT* central retinal thickness, *G&B* staging system by Gass and Blodi^4^, *SRL* superficial retinal layer, *VD* vessel density, *VDI* vessel diameter index, *DRL* deep retinal layer, *ARL* avascular retinal layer, *PNPCA* percentage of nondetectable perfused choriocapillaris area, *NA* not applicable.

### Comparison of OCT-A parameters between stable and progressive eyes

There was a significantly lower mean SRL VD in the stable (0.189 ± 0.049) compared to the progressive group (0.199 ± 0.035; p = 0.05) at baseline. Weak evidence was observed when comparing DRL VD (p = 0.08) and PNPCA (p = 0.26). A significant decrease in SRL SD in eyes with disease progression was observed between baseline and follow-up (p = 0.05). Likewise, evidence towards a decrease in DRL SD was seen in eyes with disease progression (p = 0.07). There were no statistically significant differences between stable and progressive eyes for vessel diameter index (VDI) in SRL and DRL and for all OCT-A parameters in the avascular retinal layer (ARL). Table 2 provides a detailed overview on comparison of OCT-A parameters between the stable and the progressive group.

**Table 2:** Comparison of quantitative OCT-A parameters between the stable and the progressive group.

	Stable group (mean ± SD) N = 89	Progressive group (mean ± SD) N = 33	p-value
SRL VD	0.189 ± 0.05	0.199 ± 0.035	0.05
SRL VDI (pixel)	3,586,028.96 ± 749,404.28	3,556,113.95 ± 653,486.14	0.85
SRL SD (1/pixel)	6.24E^−8^ ± 4.55E^−9^	6.28E^−8^ ± 4.02E^−9^	0.05
DRL VD	0.072 ± 0.023	0.074 ± 0.013	0.08
DRL VDI (pixel)	1,198,983.5 ± 289,278.73	1,188,723.27 ± 237,206.94	0.86
DRL SD (1/pixel)	8.46E^−8^ ± 3.17E^−9^	8.57E^−8^ ± 2.97E^−9^	0.07
ARL VD	0.056 ± 0,036	0.054 ± 0.037	0.81
ARL VDI (pixel)	1,118,591.55 ± 451,710.7	1,062,797.24 ± 419,807.59	0.95
ARL SD (1/pixel)	4.4E^−8^ ± 1.53E^−8^	4.43E^−8^ ± 1.68E^−8^	0.55
PNPCA (%)	34.46 ± 13.84	28.14 ± 7.09	0.26

*SD* standard deviation, *SRL* superficial retinal layer, *VD* vessel density, *VDI* vessel diameter index, *SD* skeleton density, *DRL* deep retinal layer, *PNPCA* percentage of nondetectable perfused choriocapillaris area.

### Prognostic relevance of OCT and OCT-A parameters

**Figure 1:**
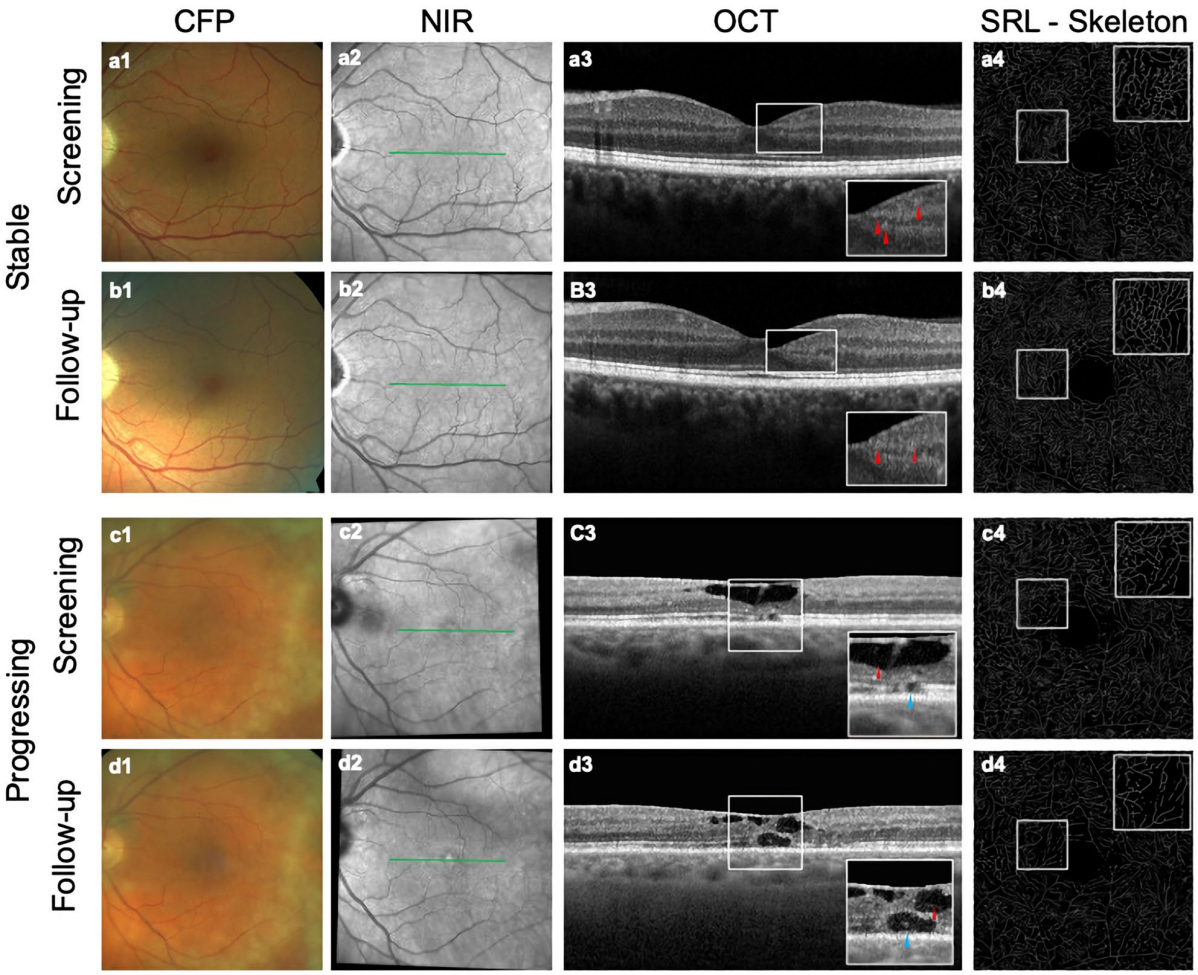
Multimodal imaging of one stable and one progressing MacTel eye. *CFP* Color fundus photography, *NIR* Near infrared reflectance image, *OCT* Optical coherence tomography, *SRL-Skeleton* Superficial retinal layer, skeletonized, *MacTel* Macular Telangiectasia Type 2, *EZ* Ellipsoid zone. a1–b4: Multimodal imaging of a left eye of a 62-year-old female MacTel patient at baseline and follow-up (18 months). a1, a2: Subtle temporal loss of retinal transparency (Stage 2). Green line in the NIR image shows the position of the OCT B-Scan. a3: Subtle alterations in the intraretinal vasculature (red arrowheads). a4: Skeletonized image of the vasculature in the superficial retinal layer. Vessels appear tightly packed and continuous. b1: Subtle temporal loss of retinal transparency (Stage 2). b2: Subtle changes in the temporal vasculature. b3: Previously observed changes in the microvasculature appear to have shifted (red arrowheads). b4: No clear changes visible compared to a4. c1–d4: Multimodal imaging of a left eye of a 65-year-old male MacTel patient at baseline and follow-up (17 months). c1: Subtle loss of retinal transparency at the temporal edge of the fovea (Stage 2). c2: Subtle reflectivity alterations at the foveal center are visible. c3: Pronounced hyporeflective intraretinal cavity (red arrow head) and subtle EZ loss (blue arrowhead). c4: Vessels appear lighter packed compared to a4. d1: Increasing loss of retinal transparency at the temporal edge of the fovea and subtle vascular changes (Stage 3). d2: Subtle reflectivity alterations at the foveal center are visible. d3: Hyporeflective cavities change in appearance (red arrowhead), increase in EZ loss. d4: Vessels appear even less densely packed and continuous than in c4.

Multivariable linear mixed effects models were used to assess the prognostic value of OCT and OCT-A parameters, CRT and BCVA on disease progression (left side of Table 3 and Supplementary Table 1) and on individual outcome measures (right side of Table 3 and Supplementary Table 2). Baseline BCVA and SRL SD were prognostic for future disease progression (p < 0.001 and p = 0.05, respectively). No statistical significance was reached for DRL VD, PNPCA and baseline CRT. Furthermore, baseline BCVA was prognostic for future BCVA (p < 0.001), EZ loss was prognostic for future CRT (p = 0.012), and baseline EZ loss, BCVA, and CRT were significant for future EZ loss (p < 0.001, p = 0.027 and p = 0.012, respectively). Figure 1 provides multimodal imaging of one stable case and one example of one progressing case.

**Table 3:** Results of four predictive models using multivariable linear mixed effects models.

Prediction of disease progression (G&B stage change)		Prediction of individual outcome measures		
Parameter	p-value	Outcome measure	Predictor	p-value
SRL VD	0.22	BCVA	Baseline BCVA	< 0.001
SRL SD	0.05		Baseline SRL VD	0.91
SRL VDI	0.13		Baseline DRL VD	0.4
DRL VD	0.87		Baseline EZ loss	0.75
			Baseline CRT	0.296
DRL SD	0.20	EZ Loss	Baseline BCVA	0.03
DRL VDI	0.70		Baseline CRT	0.012
PNPCA	0.79		Baseline SRL VD	0.45
BCVA	< 0.001		Baseline DRL VD	0.72
			Baseline EZ Loss	< 0.001
CRT	0.217	CRT	Baseline BCVA	0.4
			Baseline SRL VD	0.41
			Baseline DRL VD	0.38
			Baseline EZ Loss	0.012
			Baseline CRT	< 0.001

Left: Prediction of disease progression defined by stage switch according to grading system by Gass and Blodi. Right: Prediction of individual outcome measures. *G&B* Disease staging system by Gass and Blodi, *SRL* superficial retinal layer, *VD* vessel density, *VDI* vessel diameter index, *SD* skeleton density, *DRL* deep retinal layer, *PNPCA* percentage of nondetectable perfused choriocapillaris area, *BCVA* best corrected visual acuity, *CRT* central retinal thickness, *EZ Loss* loss of the ellipsoid zone.

## Discussion

There is a dearth of quantitative biomarkers for disease progression in MacTel. Furthermore, prognostic biomarkers for future disease progression would be of substantial value for patient and outcome measure selection for future randomized controlled clinical trials. This is the first study to analyse the prognostic value of quantitative imaging biomarkers on future disease progression in MacTel. We found SRL SD on OCT-A and EZ loss and CRT on OCT to be prognostic for disease progression.

The pathogenesis of MacTel is yet incompletely understood. Recently considered to be a primarily neurodegenerative disease, it is also characterised by vascular changes, visible in funduscopy, FLA and on OCT-A^12–16^.

Whilst several studies have reported cross-sectional OCT-A findings in MacTel, information about longitudinal alterations is limited. Demir et al. described a one year follow up of three cases^19^. They found a decrease in VD, more pronounced in the DCP compared to the SCP, which seems to be in line with the results of the study presented herein. Similar results were recently published by Hess et al.^21^.

Our data showed an association between decreased values in quantitative OCT-A parameters and future disease progression as graded by the classification system by Gass and Blodi. A recent study investigated the association between OCT-A parameters and disease staging cross-sectionally^13^. They found a decrease of quantitative OCT-A values in more advanced MacTel eyes. It has further been described that in MacTel, so-called right-angled vessels are visible, which dive from the inner retinal layers towards the choroid^14^. Our data appear to objectify these qualitative findings by indicating a decreased SRL VD and SD in progressive eyes compared to stable eyes.

Despite SRL VD and SD being decreased in progressing compared to stable eyes, only SRL SD had prognostic relevance. VD measures the percentage of the total image area occupied by blood vessels whereas SD measures the total linear length of the vessels^23^. It was reported that SD is less affected by image quality and may be superior in VD when investigating the microvasculature^23^. We hypothesize that changes in the retinal microvasculature may be the first indicators of disease progression. As these alterations are likely very subtle, only SD and not VD could be sensitive enough to visualize them.

Further, it has been shown that right-angled vessels may indicate an increased risk for NV development alluding to a similar meaning for eyes with a decreased SRL VD and SD. In contrast to previous publications on quantitative OCT-A parameters, where alterations were primarily detectable in the DRL, we found pronounced alterations in the SRL, especially when comparing progressive to stable eyes. In no case, significant differences between stable and progressive eyes were identified in the DRL. Additionally, the same cross-sectional analysis revealed the presence of flow signal in the avascular zone, solely in neovascular disease stages^13^. In this longitudinal setting, we were not able to reproduce these findings and did not find any differences in avascular layer vasculature. This might be due to not limiting our analysis to the so-called MacTel area (approx. 8° × 5° centred on the fovea), but conducting our analysis for the entire 3 mm × 3 mm field imaged by OCT-A. This decision was taken as alterations in MacTel, e.g. a more irregular and reduced CC perfusion, outside the MacTel area have been previously described^13^. Recently, an increase of CC flow voids was shown in eyes with neovascular disease stages. These findings were evident adjacent to secondary NVs but also extended beyond this area^13^. Our analysis did not show any significant alterations on CC level between progressive and stable eyes. In our understanding, this must be seen as a strength of the presented methodology, as it allows to capture MacTel features beyond the well-researched MacTel zone.

With treatment options currently emerging and novel interventional trials underway to slow disease progression, novel quantitative biomarkers are needed to identify eyes at risk for disease progression^5^. Such biomarkers are relevant for patient selection as well as for patient counselling. Our analyses provide evidence of prognostic value of quantitative OCT-A and OCT imaging biomarkers for MacTel. As MacTel has been described to have vascular components in its pathogenesis, it appears reasonable that subtle changes on the vascular level, accessible via OCT-A, precede disease progression.

As area of EZ loss associates well with functional impairment assessed by fundus controlled microperimetry and is more widely accessible, it has become the primary outcome parameter for currently ongoing interventional trials^5^. In the presented study we identify baseline BCVA and CRT values to be prognostic for future EZ loss development. Knowledge about which eyes are prone to EZ loss development may be crucial for patient selection and study result interpretation. Further, we employed a predictive model for CRT. Its general decrease over time is a known feature of the disease course^24^. In our model, follow-up CRT is best predicted by the area of EZ loss at baseline. Unlike in different other neurodegenerative retinal diseases, CRT is not equally associated with visual acuity in MacTel^24^. Decline in BCVA in MacTel is not as much due to secondary NVs but rather due to outer retinal impairment as represented by EZ loss. In addition, it is subject to speculation if even much localised outer retinal degeneration such as the punched-out defect in MacTel may also precede inner retinal degeneration, which then is revealed as general retinal thinning.

Several limitations must be considered when interpreting the results. OCT-A image acquisition is relatively sensitive and susceptible to artifacts especially in patients with impaired fixation. Before image analysis, we manually screened the captured OCT-A images to avoid the inclusion of insufficient quality data. Nonetheless, it has to be acknowledged that common features of MacTel pathology can impact the semiautomated image analysis employed in this study. Especially secondary NVs, pigment clumps or EZ loss can complicate retinal layer segmentation which may lead to imaging artifacts that can bias our results, despite careful manual layer segmentation review and correction. In addition, dense overlaying structures such as the accumulation of pigment clumps or prominent vessels may impact the analysis of underlying structures, especially the CC. Further, we did not focus our analysis on the so called MacTel area but investigated the entire 3 mm × 3 mm area imaged by OCT-A, which is a broader approach and may therefore prevent the detection of most subtle alterations. However, this may also represent a strength of the study, as this approach allows for the detection of vascular alterations even beyond the MacTel area. Strengths of our study further include the longitudinal design following the standard operation procedures of the international multicentre observational study NHOR (Natural History and Observational Registry Study, NHOR, www.mactelresearch.org), warranting for good and reliable image quality. To the best of our knowledge, the presented data set is the largest currently published longitudinal OCT-A data in MacTel. However, to increase the power of our analyses, further studies with larger sample sizes are needed. Also, to assess the clinical relevance of our findings, a longer follow-up period would be helpful.

In summary, we identified SRL VD on OCT-A and EZ loss and CRT on OCT to be prognostic for future disease deterioration. These imaging biomarkers may be considered for patient and outcome measure selection for future interventional clinical trials in patients with MacTel.

## Methods

### Patient selection

For this longitudinal, retrospective single center observational study patients were selected from the MacTel Natural History and Observation Registry (NHOR; www.mactelresearch.com) conducted at the Ophthalmology department of the University of Bonn, Bonn, Germany. Details of this study have been published elsewhere^25^. The study was approved by the local ethics committee at the University Hospital Bonn (reference number 307/17) and adhered to the declaration of Helsinki. All patients had to provide written informed consent prior to study inclusion. The diagnosis of MacTel was based on characteristic findings on funduscopy, OCT, macular pigment optical density measurements (MPOD) and fluorescence FLA^1^. Inclusion criteria were a confirmed diagnosis of MacTel and a follow up time of at least 15 months. Exclusion criteria were other relevant ocular diseases including but not limited to age-related macular degeneration, diabetic retinopathy and central serous retinopathy and the presence of optic media opacities preventing the acquisition of high-resolution retinal imaging.

### Image acquisition and disease staging

Patients underwent a full ophthalmic exam including BCVA testing, funduscopy in mydriasis, FLA (30°) centered on the fovea, spectral-domain OCT (volume scans of 25° × 30° and 10° × 15° > 97 scans) (Spectralis, HeidelbergEngineering, Heidelberg, Germany), OCT-A (Swept-Source OCT-Angiography, scans of 3 mm × 3 mm (312 horizontal A-scans, Angio mode), Zeiss PLEX Elite 9000, Carl Zeiss Meditec, Dublin, California, USA), color fundus photography (CFP 55°) centered on the fovea (Zeiss Visucam, Carl-Zeiss Meditec, Dublin California), blue fundus autofluorescence imaging (BAF), infrared imaging (IR) and a slitlamp examination at screening and at each follow-up visit. Eyes were classified using the CFP based staging system by G&B, which was used to define disease stability and disease progression^4^. Disease progression was defined as a switch from one G&B disease stage to another during the follow-up period. Disease staging was performed independently by two experienced graders (authors LG and JLR) using CFPs. In case of disagreement, a third grader arbitrated (author MWMW). G&B disease classification was used because it relies on CFP and FLA, which was not part of our outcome parameters. Stage 1 is defined the absence of findings on CFP, yet mild leakage on FLA is visible. Stage 2 is defined by the loss of retinal transparency, stage 3 shows vascular alterations like blunted vessels, stage 4 is defined by pigment clumps and stage 5 shows NVs. The recently introduced novel classification system based on multimodal imaging includes OCT findings (e.g. EZ loss) we investigated in this study^26,27^. Using this classification system instead of the G&B one would not allow for an independent assessment OCT and OCT-A parameters and their impact on disease progression.

### OCT parameters

Further parameters were retinal hyperreflective changes, defined as any hyperreflective changes located within inner or outer retinal layers and exceeding the size of small singular capillaries, subretinal neovascular membranes, hyporeflective cavities, defined as a sharply demarcated hyporreflective area within the outer and/or inner retina, presence and size of EZ loss and the CRT^28^. These were graded on OCT imaging. Neovascular membranes were defined as dense hyperreflective lesions beneath the neurosensory retina and/or the RPE^28^. Neovascular activity was defined as the presence of focal retinal thickening, subretinal and intraretinal fluid and neovascular membranes on OCT and hemorrhages on CFP^15^. To determine the size of EZ loss, images were analyzed by the EyeExplorer software (Version 6.16.7.0., HeidelbergEngineering, Heidelberg, Germany) in transverse mode and the segmentation line “PR1” (Photoreceptor 1 line, overlaying the EZ) was set as a reference for image display. The dark appearing area of EZ loss was manually demarcated using the implemented draw region tool. A detailed explanation of the measurement technique has been given elsewhere^29^. The CRT, defined as the mean thickness between the internal limiting membrane (ILM) and Bruch’s membrane (BM) was also assessed using the EyeExplorer software. Firstly, the automated segmentation for the ILM and BM was assessed and manually corrected where needed. Secondly, the OCT was displayed in the thickness map mode and the CRT value was collected for the central subfield of the early treatment diabetic retinopathy grid centered on the fovea. Qualitative parameters, i.e. the presence of EZ loss, hyperreflective changes, neovascular membranes, hyporeflective cavities, were graded similarly as the G&B disease stages. Two graders (authors LG and JLR) assessed the presence/absence of these findings independently. An arbitrator (author MWMW) was called in case of disagreement. Quantitative parameters, i.e. CRT and EZ loss area, were independently assessed by authors LG and JLR and subsequently assessed for agreement. If the difference between these measurements was < 10%, the mean was calculated and used for analysis. If the difference was > 10%, a third grader (author MWMW) conducted another independent measurement. Differences between the senior’s assessment and the previous measurements were calculated and the closest two measurements were used for mean calculation. To avoid potential biases, the graders conducted their assessments independently, on separate days, and avoided discussing their observations before their finalization.

### OCT-A parameters

After OCT-A image acquisition, images were screened by a trained grader (author LG) for image quality. Images showing insufficient quality due to artifacts, i.e. displacement artifacts, stretch artifact or vessel doubling, were excluded from any analysis^30^. OCT-A images were semiautomatically segmented into SRL, DRL, ARL and ORCC. Where required, segmentation was manually adjusted following relevant anatomical structures and the current OCT-A nomenclature^16^. Images were generated using the maximum projection algorithm of the instrument software of each individual slab within the artifact corrected volume^17,18^. Images were exported and analyzed using the FIJI software^31,32^. The superficial and deep plexus were binarized using the maximum intensity of the foveolar avascular zone as a threshold^33^. After binarization, images were skeletonized. VD, SD and VDI were calculated from the resulting images as previously described^33^. In brief, VD and SD were used to describe vascular density, while VDI was used to characterize vascular morphology. In this, VD represents a unitless proportion of the total area of pixels with detected OCT-A signal. SD measures the statistical length of the moving blood column (pixels). VDI measures the average vessel diameter in pixels, essentially by dividing white pixels of the binarized OCT-A image by the white pixels of the skeletonized OCT-A image^33^. Quantitative analysis was conducted for the entire 3 × 3 mm OCT-A scan. The CC-slab was analyzed for mean signal intensity, kurtosis and percentage of nondetectable perfused CC-area PNPCA as described elsewhere^34,35^. Briefly, images were binarized using the Phansalkar method as originally described by R. Spaide et al.^36^ with a radius of 50 pixels as this has shown to be sensitive for greater flow alterations in ORCC^13,35,37^. Subsequently, the “Analyze particles” command of FIJI was used to count and assess the size of all areas containing an absence of flow information (“flow voids”) as a percentage of the PNPCA.

### Statistical analysis

Statistical analyses were performed using R (Version 4.3.1)^38^. Linear mixed effects models, adjusting for patients’ age, sex and the nested data structure of two eyes within one patient, were utilized to assess differences in OCT-A parameters comparing patients with a stable disease course and patients with disease progression. For these linear mixed effects models, the OCT-A parameters (as continuous variables) were the outcome parameters (dependent variable). The stability or progression of the respective disease stage is treated as an independent variable. In the first step, we conducted a univariate regression analysis to assess for differences in the aforementioned parameters between eyes displaying disease progression and stable eyes. OCT-A parameters, which showed at least a trend (p < 0.1) to be different in stable compared to progressive eyes (SRL VD, SRL SD, DRL VD, DRL SD), and OCT (EZ loss, CRT), parameters as well as BCVA were employed in a multivariable predictive model to assess their impact on individual outcome measures. Further, we established a predictive model to analyze the impact of baseline OCT parameters (EZ loss, CRT), OCT-A parameters (VD, SD, VDI, PNPCA), and BCVA on future disease progression. We fitted four models, where disease progression (G&B stage change), future BCVA, EZ loss, and CRT each served as outcome variables. Baseline OCT and OCT-A parameters, in addition to baseline BCVA were considered as potential prognostic variables, respectively. p values < 0.05 were considered significant.

### Supplementary Information

Supplementary Table 1.

Supplementary Table 2.

## Data Availability

Original data is available from the corresponding author upon reasonable request.

## References

[1] Charbel Issa, P. *et al.* Macular telangiectasia type 2. *Prog. Retin. Eye Res.* **34**, 49–77 (2013). 23219692 10.1016/j.preteyeres.2012.11.002 PMC3638089

[2] Peto, T. *et al.* Correlation of clinical and structural progression with visual acuity loss in Macular Telangiectasia Type 2. *Retina* **38**, S8–S13 (2018). 28505012 10.1097/IAE.0000000000001697 PMC8326288

[3] Runkle, A. P. *et al.* OCT angiography and ellipsoid zone mapping of macular telangiectasia type 2 from the AVATAR study. *Investig. Ophthalmol. Vis. Sci.* **58**, 3683–3689 (2017). 28727884 10.1167/iovs.16-20976 PMC5518977

[4] Gass, J. D. M. & Blodi, B. A. Idiopathic juxtafoveolar retinal telangiectasis: Update of classification and follow-up study. *Ophthalmology* **100**, 1536–1546 (1993). 8414413 10.1016/S0161-6420(93)31447-8

[5] Chew, E. Y. *et al.* Effect of ciliary neurotrophic factor on retinal neurodegeneration in patients with Macular Telangiectasia Type 2: A randomized clinical trial. *Ophthalmology* **126**, 540–549 (2019). 30292541 10.1016/j.ophtha.2018.09.041 PMC8365464

[6] Mandal, S., Venkatesh, P., Abbas, Z., Vohra, R. & Garg, S. Intravitreal bevacizumab (Avastin) for subretinal neovascularization secondary to type 2A idiopathic juxtafoveal telangiectasia. *Graefe’s Arch. Clin. Exp. Ophthalmol.* **245**, 1825–1829 (2007). 17345090 10.1007/s00417-007-0567-8

[7] Roller, A. B. *et al.* Intravitreal bevacizumab for treatment of proliferative and nonproliferative type 2 idiopathic macular telangiectasia. *Retina* **31**, 1848–1855 (2011). 21610563 10.1097/IAE.0b013e31820d3feb

[8] Narayanan, R. *et al.* Efficacy of anti-vascular endothelial growth factor therapy in subretinal neovascularization secondary to (2011). 10.1097/IAE.0b013e3182625c1d 22990322

[9] Spaide, R. F., Fujimoto, J. G. & Wahee, N. Optical coherence tomography angiography. *Retina* **62**, 1757 (2015). 10.1097/IAE.0000000000000881 PMC4710360 26502006

[10] Choi, W. J. *et al.* Choriocapillaris and choroidal microvasculature imaging with ultrahigh speed OCT angiography. *PLoS One* **8**, e81499 (2013). 24349078 10.1371/journal.pone.0081499 PMC3859478

[11] Kim, D. Y. *et al.* Optical imaging of the chorioretinal vasculature in the living human eye. *Proc. Natl. Acad. Sci. U. S. A.* **110**, 14354–14359 (2013). 23918361 10.1073/pnas.1307315110 PMC3761584

[12] Charbel Issa, P. *et al.* Macular telangiectasia type 2. *Progr. Retin. Eye Res.* **34**, 49–77. 10.1016/j.preteyeres.2012.11.002 (2013). 10.1016/j.preteyeres.2012.11.002 PMC3638089 23219692

[13] Tzaridis, S. *et al.* Quantification of retinal and choriocapillaris perfusion in different stages of Macular Telangiectasia Type 2. *Investig. Ophthalmol. Vis. Sci.* **60**, 3556–3562 (2019). 31415079 10.1167/iovs.19-27055

[14] Tzaridis, S. *et al.* Right-angled vessels in macular telangiectasia type 2. *Br. J. Ophthalmol.* **105**, 1289–1296 (2021). 30808615 10.1136/bjophthalmol-2018-313364 PMC8380913

[15] Tzaridis, S., Hess, K., Friedlander, M. & Holz, F. G. Optical coherence tomography-angiography for monitoring neovascularisations in macular telangiectasia type 2. *Br. J. Ophthalmol.* **105**, 735–740 (2021). 32513667 10.1136/bjophthalmol-2020-316021

[16] Roisman, L. & Rosenfeld, P. J. Optical coherence tomography angiography of Macular Telangiectasia Type 2. *OCT Angiogr. Retin. Macular Dis.*10.1159/000442807 (2016). 10.1159/000442807 27022942

[17] Toto, L. *et al.* Multimodal imaging of macular telangiectasia type 2: Focus on vascular changes using optical coherence tomography angiography. *Investig. Ophthalmol. Vis. Sci.* **57**, OCT268–OCT276 (2016). 27409482 10.1167/iovs.15-18872

[18] Dogan, B., Erol, M. K., Akidan, M., Suren, E. & Akar, Y. Retinal vascular density evaluated by optical coherence tomography angiography in macular telangiectasia type 2. *Int. Ophthalmol.* **39**, 2245–2256 (2019). 30607862 10.1007/s10792-018-01060-x

[19] Demir, S. T., Güven, D. & Karatas, M. E. Evaluation of 1-year follow-up results of macular telangiectasia type 2 cases by optical coherence tomography angiography (MacTel 2) were evaluated. The 3X3 mm OCTA imaging was performed. **9**, 1–5 (2019). 10.3205/oc000118 PMC6734514 31531275

[20] Venkatesh, R. *et al.* The preproliferative stage in type 2 macular telangiectasia (MacTel type 2). *Graefe’s Arch. Clin. Exp. Ophthalmol.* **260**, 121–132 (2022). 34410484 10.1007/s00417-021-05371-1

[21] Hess, K., Charbel Issa, P., Holz, F. G. & Tzaridis, S. Morphological characteristics preceding exudative neovascularisation secondary to macular telangiectasia type 2. *Br. J. Ophthalmol.* **106**, 1736–1741 (2021). 34167944 10.1136/bjophthalmol-2020-318470

[22] Goerdt, L. *et al.* Relative Ellipsoid Zone Reflectivity in Macular Telangiectasia Type 2. *Investig. Ophthalmol. Vis. Sci.* **64**, 21 (2023). 10.1167/iovs.64.10.21 PMC10362918 37462978

[23] Angeli, O. *et al.* Qualitative and quantitative comparison of two semi-manual retinal vascular density analyzing methods on optical coherence tomography angiography images of healthy individuals. *Sci. Rep.* **13**, (2023). 10.1038/s41598-023-44234-z PMC10562399 37813968

[24] Charbel Issa, P., Helb, H. M., Holz, F. G. & Scholl, H. P. N. Correlation of macular function with retinal thickness in nonproliferative type 2 idiopathic Macular Telangiectasia. *Am. J. Ophthalmol.* **145**, 169–176 (2008). 17981256 10.1016/j.ajo.2007.08.028

[25] Clemons, T. E. *et al.* Baseline characteristics of participants in the natural history study of Macular Telangiectasia (MacTel) MacTel Project Report No. 2. *Ophthal. Epidemiol.* **17**, 66–73 (2010). 10.3109/09286580903450361 PMC8329604 20100102

[26] Wu, Y. *et al.* Developing a continuous severity scale for Macular Telangiectasia Type 2 using deep learning and implications for disease grading. *Ophthalmology* **131**, 219–226 (2024). 37739233 10.1016/j.ophtha.2023.09.016 PMC10841914

[27] Chew, E. Y. *et al.* Macular Telangiectasia Type 2: A classification system using multimodal imaging MacTel project report number 10. *Ophthalmol. Sci.* **3**, (2023). 10.1016/j.xops.2022.100261 PMC9944556 36846105

[28] Tzaridis, S. *et al.* Hyper-reflectivity on optical coherence tomography in macular telangiectasia type 2. *Retina*. (2021). 10.1097/IAE.0000000000003031 33230067

[29] Heeren, T. F. C. *et al.* Longitudinal correlation of ellipsoid zone loss and functional loss in Macular Telangiectasia Type 2. *Retina*10.1097/IAE.0000000000001715 (2017). 10.1097/IAE.0000000000001715 PMC8326028 28541959

[30] Spaide, R. F., Fujimoto, J. G. & Waheed, N. K. Image artifacts in optical coherence tomography angiography. *Retina*. **35** http://journals.lww.com/retinajournal (2015). 10.1097/IAE.0000000000000765 PMC4712934 26428607

[31] Schindelin, J. *et al.* Fiji: An open-source platform for biological-image analysis. *Nat. Methods* **9**, 676–682 (2012). 22743772 10.1038/nmeth.2019 PMC3855844

[32] Schneider, C. A., Rasband, W. S. & Eliceiri, K. W. NIH image to ImageJ: 25 years of image analysis. *Nat. Methods* **9**, 671–675 (2012). 22930834 10.1038/nmeth.2089 PMC5554542

[33] Kim, A. Y. *et al.* Quantifying retinal microvascular changes in uveitis using spectral-domain optical coherence tomography angiography. *Am. J. Ophthalmol.* **171**, 101–112 (2016). 27594138 10.1016/j.ajo.2016.08.035 PMC5115969

[34] Spaide, R. F. & Curcio, C. A. Evaluation of segmentation of the superficial and deep vascular layers of the retina by optical coherence tomography angiography instruments in normal eyes. *JAMA Ophthalmol.* **135**, 259–262 (2017). 28097291 10.1001/jamaophthalmol.2016.5327

[35] Wintergerst, M. W. M. *et al.* Optical coherence tomography angiography in intermediate uveitis. *Am. J. Ophthalmol.* **194**, 35–45 (2018). 30026083 10.1016/j.ajo.2018.06.023

[36] Spaide, R. F. Choriocapillaris flow features follow a power law distribution: Implications for characterization and mechanisms of disease progression. *Am. J. Ophthalmol.* **170**, 58–67 (2016). 27496785 10.1016/j.ajo.2016.07.023

[37] Wintergerst, M. W. M. *et al.* Vessel density on optical coherence tomography angiography is prognostic for future disease course in intermediate uveitis. *Sci. Rep.* **14**, (2024). 10.1038/s41598-023-49926-0 PMC10844199 38317017

[38] Team, R. C. *R: A Language and Environment for Statistical Computing.* 2003–2005 (2022).

