# Supplementary material for: Skeleton density and ellipsoid zone loss are prognostic for progression in Macular Telangiectasia Type 2
Source: Sci Rep. 2024 Jul 27;14:17328. doi: 10.1038/s41598-024-67801-4 (PMC11283486; doi:10.1038/s41598-024-67801-4)
Supplement: Supplementary file 1 — Supplementary Table 1. [file 41598_2024_67801_MOESM1_ESM.docx]

**Supplementary table 1:**

Results of the multivariable mixed effects model to assess the prognostic value of OCT-A parameters, BCVA and CRT on disease progression.

| Coefficients | Estimate | Std. error | z value | p-value |
| --- | --- | --- | --- | --- |
| SRL VD | 0.009 | 0.008 | 1.246 | 0.22 |
| SRL SD | -2.375E-9 | -1.225E-9 | -1.940 | 0.05 |
| SRL VDI | 233381 | 152599 | 1.529 | 0.13 |
| DRL VD | 0.001 | 0.005 | 0.168 | 0.87 |
| DRL SD | -1.2E-9 | 9.249E-10 | -1.298 | 0.20 |
| DRL VDI | 23568 | 61922 | 0.381 | 0.70 |
| PNPCA | -0.96 | 3.983 | -0,233 | 0.79 |
| BCVA | 0.993 | 0.193 | 5.142 | < 0.001 |
| CRT | -0.002 | 0.001 | -1.242 | 0.217 |

Abbreviations: SRL = superficial retinal layer, VD = vessel density, VDI = vessel diameter index, SD = skeleton density, DRL = deep retinal layer, PNPCA = percentage of nondetectable perfused choriocapillaris area, BCVA = best corrected visual acuity, CRT = central retinal thickness
