# Supplementary material for: Skeleton density and ellipsoid zone loss are prognostic for progression in Macular Telangiectasia Type 2
Source: Sci Rep. 2024 Jul 27;14:17328. doi: 10.1038/s41598-024-67801-4 (PMC11283486; doi:10.1038/s41598-024-67801-4)
Supplement: Supplementary file 2 — Supplementary Table 2. [file 41598_2024_67801_MOESM2_ESM.docx]

**Supplementary table 2:**

Results of the multivariable mixed effects models to assess the prognostic value of OCT-A parameters, BCVA, CRT and the presence of EZ loss on individual outcome measures.

| Outcome parameter | Coefficients | Estimate | Std. error | Z value | P |
| --- | --- | --- | --- | --- | --- |
| BCVA | SRL VD | 0.082 | 0.753 | 0.109 | 0.91 |
|  | DRL VD | -0.892 | 1.055 | -0.845 | 0.4 |
|  | Baseline BCVA | 0.939 | 0.089 | 10.573 | < 0.001 |
|  | EZ loss | -0.026 | 0.081 | -0.32 | 0.74 |
|  | CRT | 5.168E-4 | 4.921E-4 | 1.05 | 0.296 |
| EZ loss | SRL VD | -9.121E-1 | 1.204 | -0.757 | 0.451 |
|  | DRL VD | -6.717E-1 | 1.847 | -0.364 | 0.717 |
|  | BCVA | 2.165E-1 | 9.68E-2 | 2.237 | 0.03 |
|  | Baseline EZ loss | 1.186 | 0.055 | 21.45 | < 0.001 |
|  | CRT | -1.735E-3 | 6.767E-4 | -2.564 | 0.012 |
| CRT | SRL VD | 123.404 | 150.874 | 0.818 | 0.415 |
|  | DRL VD | 204.563 | 233.25 | 0.877 | 0.383 |
|  | BCVA | 8.264 | 9.876 | 0.837 | 0.404 |
|  | EZ loss | -33.767 | 13.239 | -2.55 | 0.123 |
|  | Baseline CRT | 0.75 | 0.066 | 11.372 | < 0.001 |

Abbreviations: SRL = superficial retinal layer, VD = vessel density, DRL = deep retinal layer, BCVA = best corrected visual acuity, CRT = central retinal thickness, EZ = ellipsoid zone
